# How to care for and clean optical surfaces

**Published:** 2010-12

**Authors:** Ismael Cordero

**Affiliations:** Senior Clinical Engineer, ORBIS International, 520 8th Avenue, 11th Floor, New York, NY 10018, USA.

**Figure:**
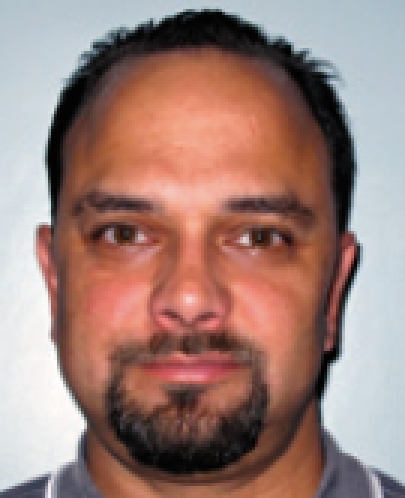


Many ophthalmic devices have optical components such as windows, lenses, mirrors, filters, and prisms; even very small irregularities (such as scratches) can cause unwanted scattering of light which reduces quality. The surfaces of lenses, prisms, and windows are often coated with an antireflective layer to prevent loss of light due to reflection. Mirrors have a highly reflecting coating to get maximum reflection of light. Filters have coatings to cut out undesired wavelengths. The coatings are very thin and delicate and can be damaged by improper handling and cleaning.

By following these suggestions, you will help ensure that all of the optical surfaces in your eye care equipment perform optimally.

## General care

- Place a dust cover over eye care equipment when not in use.
- Always replace the lens caps, if available, when not in use.
- Keep eye care equipment in an environment that is not humid; this will prevent the growth of fungus on optical components (see Issue 73, page 37).
- Repeated cleaning will wear out the surface coating described earlier and the property of the surface may change. It is better to protect optical components from dust, stain and fungus.

## Before you clean

- If the optical component is not dirty, do not clean it.
- First, read the manufacturer's instructions.
- Laser optics should only be cleaned by trained, qualified specialists.

## What you will need

**Figure 1.**
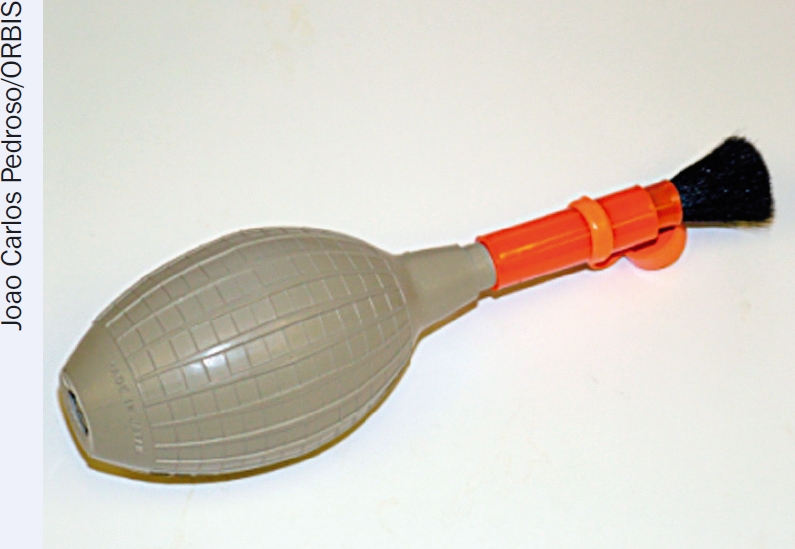
A dust blower can be found at most photography shops. Some blowers, such as the one pictured, have a built-in lens brush.

**Figure 2.**
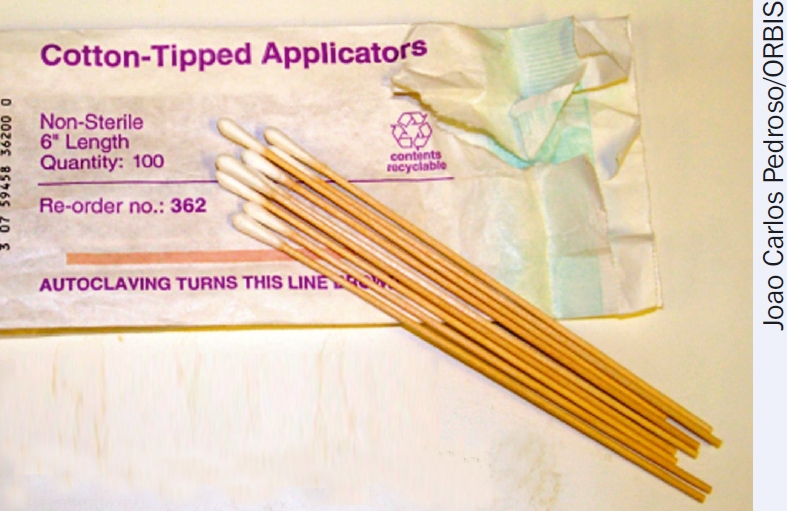
Non-sterile, medical-grade cotton swabs

- dust blower (Figure 1)
- lens brush, which is sometimes attached to the dust blower (Figure 1)
- lint-free lens tissue (available in photography shops)
- optical cleaning solutions (see below)
- lint-free cotton gloves
- bamboo tweezers
- cotton swabs (Figure 2). Use non-sterile, medical-grade cotton swabs, with degreased fibres that will not release lint. If these are not available, fresh cotton swabs can be prepared with wooden sticks and medical-grade cotton.

Joao Carlos Pedroso/ORBIS

Joao Carlos Pedroso/ORBIS

## Optical cleaning solutions

First consult the manufacturer. If this is not possible, commercial lens-cleaning solutions sold in camera shops can be used for most optical cleaning. Otherwise try the following, going from weakest (1) to strongest (4) until the surface is clean:

1. Distilled water
2. A water-based solution: 1 part mild, neutral detergent to 19 parts distilled water
3. A mixture of 60% acetone and 40% methanol (not for use on plastic lenses)
4. Isopropyl alcohol (90% purity). Note: slow evaporation can leave drying marks on the surface.

## Tips for cleaning

**Figure 3.**
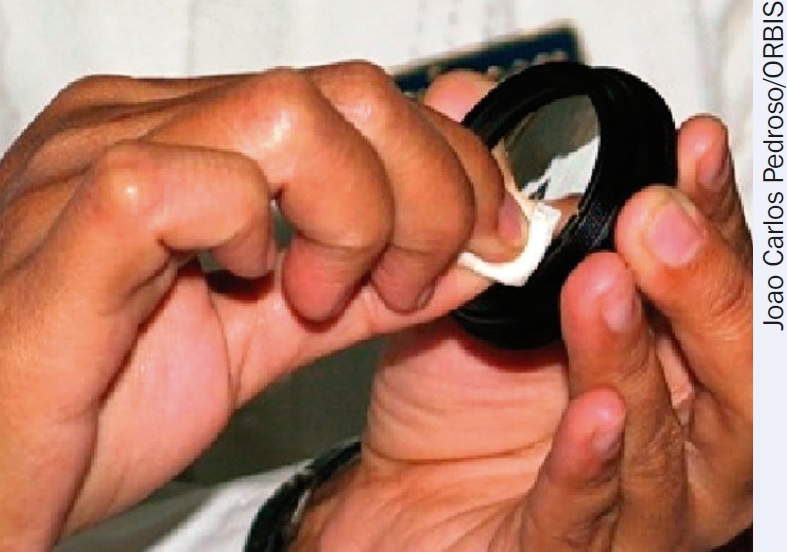
Always handle loose optical components by their edges. Clean round optical surfaces from the centre to the edge in a widening spiral.

Do not touch optical surfaces with your bare fingers, since they leave behind grease and moisture marks that are hard to remove. Wear cotton, lint-free gloves if available and hold loose optical components by their edge (Figure 3).

Joao Carlos Pedroso/ORBIS

- Be careful of sharp instruments, including your fingernails, near optical surfaces. Use wooden, bamboo, or plastic implements instead. Always place optical components on a soft surface to avoid scratches.
- Do not apply optical cleaning solutions directly onto the optical components of a device, since these can enter the machine and cause spots on other lenses or otherwise damage the device. Instead, apply the cleaning solution to the lens paper or swab first.
- When removing stains, avoid excess pressure since this can remove the delicate surface coating.

## Steps

1. Always remove dust first! Dust particles can produce scratches. Never wipe dust off optical surfaces, especially when they are dry. First use a dust blower to remove dust. Use a camel hair brush, sometimes included as part of the blower (Figure 1), to remove any dust that sticks to the surface.
2. To remove stains, use a lint-free cotton swab or a lens-cleaning tissue dipped in the optical cleaning solution. For round surfaces, move the swab or tissue in a circular path, starting from the centre and going in a widening spiral towards the edge (Figure 3). For rectangular surfaces, use repeated strokes parallel to each other and in the same direction until you have covered the surface. Repeat this step, using a fresh swab every time, until the stain is no longer visible at any angle under a bright light. If cotton lint is left on the component, remove it using the blower or a clean lens brush.
3. To remove stains on plastic components, use optical cleaning solution number 2 (the water-based solution). Do not use other cleaning solutions on plastic, unless indicated by the manufacturer, because they can permanently cloud the surface.
4. To remove fungus, use an optical fungicide. If unavailable, use surgical scrub soap.

**New Series**

This is the second instalment in a series on practical equipment care and maintenance.

